# GC Snakes: An Efficient and Robust Segmentation Model for Hot Forging Images

**DOI:** 10.3390/s24154821

**Published:** 2024-07-25

**Authors:** Xiaoyu Pan, Delun Wang

**Affiliations:** School of Mechanical Engineering, Dalian University of Technology, Dalian 116024, China; jonnypanxy@mail.dlut.edu.cn

**Keywords:** active contour model, grayscale surface properties, image segmentation, machine vision, real-time measurement, stereo vision processing

## Abstract

Machine vision is a desirable non-contact measurement method for hot forgings, as image segmentation has been a challenging issue in performance and robustness resulting from the diversity of working conditions for hot forgings. Thus, this paper proposes an efficient and robust active contour model and corresponding image segmentation approach for forging images, by which verification experiments are conducted to prove the performance of the segmentation method by measuring geometric parameters for forging parts. Specifically, three types of continuity parameters are defined based on the geometric continuity of equivalent grayscale surfaces for forging images; hence, a new image force and external energy functional are proposed to form a new active contour model, Geometric Continuity Snakes (GC Snakes), which is more percipient to the grayscale distribution characteristics of forging images to improve the convergence for active contour robustly; additionally, a generating strategy for initial control points for GC Snakes is proposed to compose an efficient and robust image segmentation approach. The experimental results show that the proposed GC Snakes has better segmentation performance compared with existing active contour models for forging images of different temperatures and sizes, which provides better performance and efficiency in geometric parameter measurement for hot forgings. The maximum positioning and dimension errors by GC Snakes are 0.5525 mm and 0.3868 mm, respectively, compared with errors of 0.7873 mm and 0.6868 mm by the Snakes model.

## 1. Introduction

Forging is a conventional processing method for producing key components of large-scale industrial equipment. These hot forgings have large dimensions and high temperatures and are processed in harsh industrial conditions, which makes real-time measurement extremely difficult and risky during the forging process [1,2]. As necessary measurement parameters in the forging process, geometric parameters such as the size and position of hot forgings are usually measured by contact measurement methods, even by manual work, which makes it hard to guarantee the accuracy, efficiency, and reliability of measurement. Therefore, non-contact measurement methods with high precision and robustness should have significant applications in the processing of forgings.

In regard to the non-contact measurement methods, machine vision has become one of the most widely used measurement methods due to its satisfying accuracy and efficiency. Divided into active and passive visual measurements, visual measurement involves main steps such as image segmentation, feature extraction, matching, and reconstruction; among the steps, image segmentation has a significant impact on the measurement results because the target contour determined by the segmentation directly affects the accuracy for feature extraction and matching, which makes it one of the core issues in visual measurement.

Existing image segmentation algorithms utilize the differentiation by regions in the image, such as similarity within the same region, connectivity between pixels within a region, and discontinuity between the target area and background. Classified by varieties in algorithm mechanism, the existing image segmentation methods include traditional methods such as threshold-based segmentation [3,4,5,6], edge-based segmentation [7,8,9], region-based segmentation [10,11,12,13,14], clustering-based segmentation [15,16,17,18], random-field-based segmentation [19,20,21,22,23], genetic-algorithm-based segmentation [24], etc., as well as methods based on or combined with deep learning [25,26,27,28,29,30,31,32,33,34]. The traditional methods are generally simple and easy to accomplish and thus are suitable for specific categories of images to achieve good segmentation performance. However, their simplicity also leads to limitations of poor robustness, sensitivity to image noise, and a deficiency of boundary information between regions, which makes traditional methods not suitable for forging images. On the other hand, deep learning methods segment images by making predictions from datasets to have preferable accuracy and adaptability, but their dependence on the datasets also results in long operation times and high computational costs. As an essential issue in real-time visual measuring, the segmentation for forging images focuses on the boundary information between parts and backgrounds to pursue geometric features of individual parts; meanwhile, the segmentation processing requires high efficiency and adaptability, so the general image segmentation methods have limitations for real-time forging image processing and are not compatible for industrial productions. In view of the particularity of the real-time segmentation of forging images, segmentation methods with high efficiency and stability have been proposed. Dworkin S and Nye T [35] performed binary processing on the forging image to remove unnecessary details and then searched for target boundaries from the left side between the top and bottom of the forging part area. Nie S et al. [36] and Li Z [37] extracted target boundaries for segmentation by analyzing the continuity of the grayscale distribution curve of the forging image. These methods extract the outer contour by the local gradients of the grayscale cross-section and are simple and fast but are prone to confusing low grayscale textures with the background. Therefore, these segmentation methods also have unsatisfactory accuracy and thus are not suitable for the accurate extraction of the target contour for forging parts. Wang et al. [1] combined geometric continuity edges with active contour models [38,39,40,41,42,43] to automatically select initial control points for the Snakes model to achieve good segmentation performance and computational efficiency. However, the processing conditions of hot forgings are highly uncertain and may generate significant differences in the final forging temperature and textures near the contour for forging parts, which results in remarkable differences in grayscale distribution among forging images under the same image acquisition conditions. Notably, these grayscale distribution differences may have caused the failure of the methods in [1] because in existing active contour models, the contour converges through image forces solely dominated by the bidirectional gradient, which results in weak image forces for images with inadequate grayscale differences between the target and background, making it difficult for active contours to converge to the target contour. Therefore, in response to the shortcomings of existing methods, it is important to improve the segmentation performance and robustness for the real-time processing of hot forging images.

As mentioned above, although existing image segmentation methods can theoretically achieve the function of forging image segmentation, there still exist limitations such as poor robustness, low computational efficiency, and high hardware requirements when applied to the real-time measurement of forgings. Therefore, it has always been an important issue to achieve hot forging image segmentation efficiently and stably in response to the problem that existing image segmentation algorithms may fail due to varieties of sizes and thermal radiation intensities for hot forgings. Hence, this paper proposes a new image force, as well as an external energy functional based on the geometric properties of the grayscale surface of forging images, by which an efficient and robust active contour model, namely the Geometric Continuity Snakes model (GC Snakes model) is proposed to form an image segmentation method suitable for the online real-time measurement of forging images.

The main contributions of this paper are as follows:

(1) Three types of continuity parameters are proposed based on the geometric properties of the equivalent grayscale surface of forging images, by which continuity feature edges are defined from the continuity edges;
(2) Based on the continuity parameters of forging images, geometric continuity external energy functional and image forces are proposed, by which the energy functional of the GC Snakes active contour is defined to form the GC Snakes model;
(3) The generating method for initial control points of the GC Snakes model is proposed from the continuity feature edges, by which an image segmentation method suitable for online real-time measurement is proposed for geometric parameters of hot forgings.

This paper is organized as follows: in Section 2, three types of continuity parameters are defined based on the geometric properties of equivalent grayscale surfaces, and geometric continuity edges and feature edges are provided accordingly. In Section 3, a new external energy functional and image force is proposed based on the continuity parameters of grayscale surfaces to form the GC Snakes model and the corresponding image segmentation method; Section 4 introduces the verification experiment for the GC Snakes model by measuring the positioning and dimension for a forging test part. Finally, concluding remarks and future research scopes are given in Section 5.

## 2. Grayscale Surface Continuity and Feature Edges

In the process of vision measurement, hot forgings have conspicuous grayscale and adequate textures to form features compared with the background; thus, passive visual measurement is an appropriate selection for forging parts. In this section, passive visual images of forgings are converted to discrete grayscale surfaces, and the geometric continuity of the grayscale surface is investigated to propose continuity parameters, continuity edges, and corresponding feature edges.

### 2.1. Grayscale Continuity and Continuity Parameters

**Figure 1:**
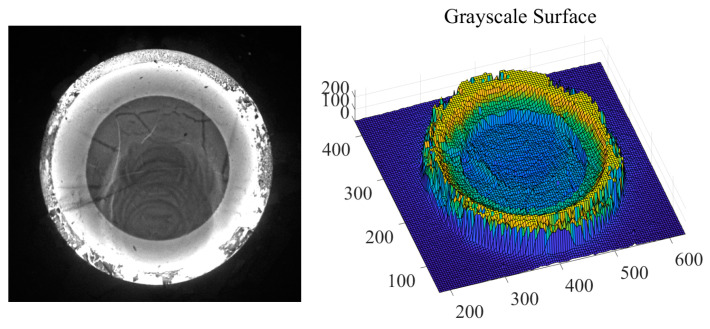
Passive forging image and the discrete grayscale surface.

Suppose the resolution of the passive visual image is *M*_0_ × *N*_0_; the grayscale image of the hot forging can be transformed into a discrete grayscale surface by taking each pixel as coordinates of *x* and *y* while taking the grayscale *h* ∈ [0, 255] of the pixel as a coordinate of *z*, shown in Figure 1. The grayscale surface Σ can be expressed as

(1) $$\left\{\begin{array}{l} z=z(x,y)=h(i,j)\\ x=i,i=1,2,\cdots ,N_{0}\\ y=j,j=1,2,\cdots ,M_{0} \end{array}\right.$$

As a most general geometric property, the smoothness of grayscale surfaces can be reflected by geometric continuity, which includes the zero-order, first-order, and second-order geometric continuity of grayscale surfaces. The zero-order geometric continuity of a grayscale surface, denoted as G^0^ continuity, requires the positions of the two constitutive sub-surfaces to be continuous, ensuring that the sub-surfaces are in contact to have no gaps. The first-order geometric continuity of a grayscale surface, denoted as G^1^ continuity, requires the tangent planes of the two constitutive sub-surfaces to be continuous, ensuring that the two sub-surfaces have a common tangent plane or surface normal at every point along the connecting line. The second-order geometric continuity of a grayscale surface, denoted as G^1^ continuity, requires the curvature of the two constitutive sub-surfaces to be continuous, ensuring that the two sub-surfaces have common normal curvature in all directions along the connecting line. According to the definition of grayscale surface continuity, this paper refers to the grayscale gradient, surface normal vector, and normal curvature gradient in each direction as G^0^, G^1^, and G^2^ continuity parameters, respectively.

**Figure 2:**
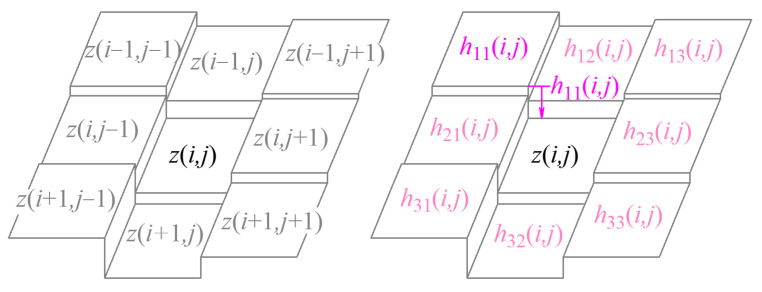
Discrete grayscale surface and G^0^ continuity parameter.

The G^0^, G^1^, and G^2^ continuity parameters are represented below. The G^0^ continuity parameter concentrates on the eight-directional grayscale gradient in the eight-connected neighborhood of the reference pixel. On the discrete grayscale surface Σ, the grayscale gradients along the eight directions in the neighborhood at pixel (*i*, *j*), except the boundary points of the image, can be represented as the grayscale difference between point (*i*, *j*) and the adjacent eight pixels, as shown in Figure 2, which are defined as

(2) $$\left\{\begin{array}{l} h_{11}(i,j)=z(i,j)-z(i-1,j-1)\\ h_{12}(i,j)=z(i,j)-z(i-1,j)=g_{x}(i,j)\\ h_{13}(i,j)=z(i,j)-z(i-1,j+1)\\ h_{21}(i,j)=z(i,j)-z(i,j-1)=g_{y}(i,j)\\ h_{23}(i,j)=z(i,j)-z(i,j+1)\\ h_{31}(i,j)=z(i,j)-z(i+1,j-1)\\ h_{32}(i,j)=z(i,j)-z(i+1,j)\\ h_{33}(i,j)=z(i,j)-z(i+1,j+1) \end{array}\right.$$

**Figure 3:**
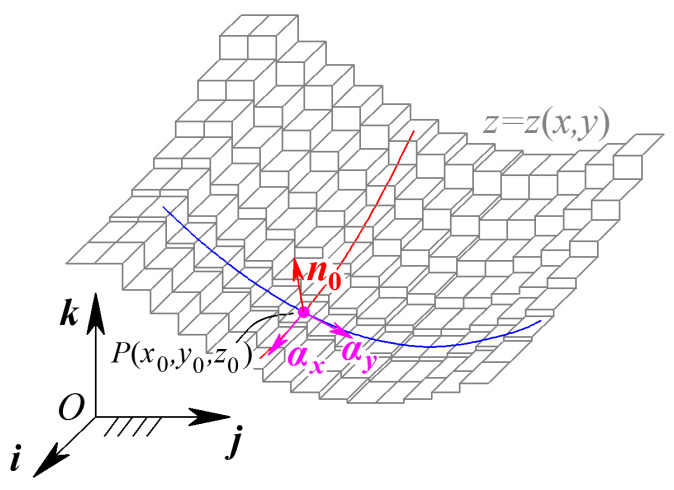
Normal vector of grayscale surface.

The G^1^ continuity parameter concentrates on the surface normal vector direction for pixels in the eight-connected neighborhood of the reference pixel. On the discrete grayscale surface Σ, the normal vector at point (*x*_0_, *y*_0_) can be represented as the cross-product of tangent vector ***α****_x_* on the x-parametric curve *Γ_x_* and tangent vector ***α****_y_* on the y-parametric curve *Γ_y_*, shown in Figure 3. Thus, the unit normal vector at point (*x*_0_, *y*_0_) can be expressed as

(3) $$\left\{\begin{array}{l} \alpha_{x}={\left[\begin{array}{ccc} 1 & 0 & \frac{\partial z}{\partial x} \end{array}\right]}_{\left(x_{0},y_{0}\right)}^{T}={\left[\begin{array}{ccc} 1 & 0 & g_{x} \end{array}\right]}_{\left(x_{0},y_{0}\right)}^{T}\\ \alpha_{y}={\left[\begin{array}{ccc} 0 & 1 & \frac{\partial z}{\partial y} \end{array}\right]}_{\left(x_{0},y_{0}\right)}^{T}={\left[\begin{array}{ccc} 1 & 0 & g_{y} \end{array}\right]}_{\left(x_{0},y_{0}\right)}^{T}\\ n_{0}(x_{0},y_{0})=\alpha_{x(x_{0},y_{0})}\times \alpha_{y(x_{0},y_{0})}={\left[-g_{x},-g_{y},1\right]}_{\left(x_{0},y_{0}\right)}^{T}\\ n(x_{0},y_{0})=\frac{n_{0}(x_{0},y_{0})}{\left|n_{0}(x_{0},y_{0})\right|} \end{array}\right.$$

For the reference pixel (*i*, *j*) on the equivalent grayscale surface, the grayscale gradients *g_x_* and *g_y_* can be represented by the G^0^ order continuity parameters. Substituting Equation (2) into Equation (3), the unit normal vector at pixel (*i*, *j*) on the equivalent grayscale surface can be expressed as

(4) $$\left\{\begin{array}{l} n_{0}(i,j)={\left[-h_{12}(i,j),-h_{21}(i,j),1\right]}^{T}\\ n(i,j)=\frac{n_{0}(i,j)}{\left|n_{0}(i,j)\right|} \end{array}\right.$$

**Figure 4:**
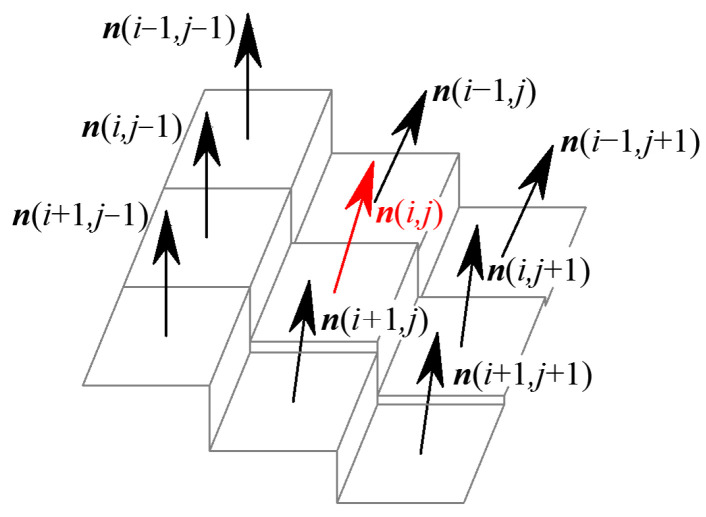
G^1^ continuity parameter.

Thus, the G^1^ continuity parameter at pixel (*i*, *j*), namely the surface normal vector direction within the eight-connected neighborhood, is shown in Figure 4.

**Figure 5:**
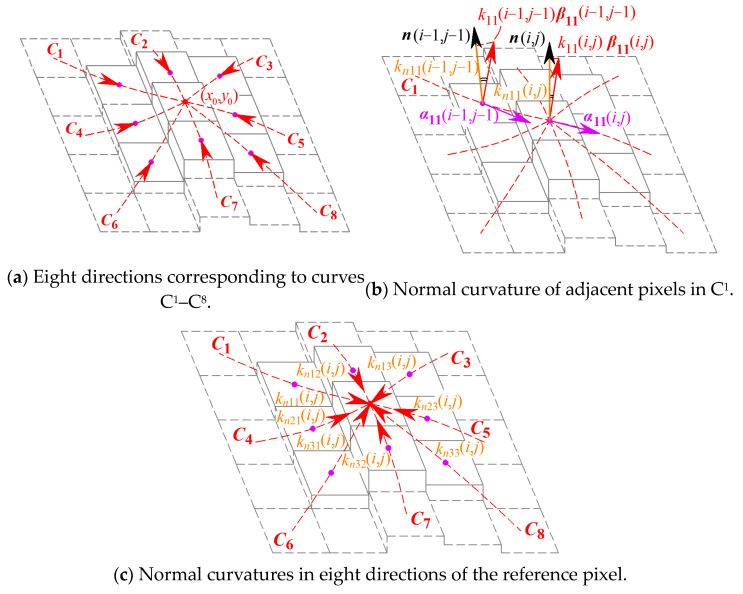
Normal curvatures of the reference pixel in eight directions.

The G^2^ continuity parameter concentrates on the normal curvatures of the reference point and those in the eight-connected neighborhood along the respective directions. If ***r*** = ***r***(*x*, *y*) is the vector equation for the discrete grayscale surface Σ, the unit tangent vector ***α***_0*C*_ and the principal normal vector ***β***_0*C*_ at point *P*(*x*_0_, *y*_0_) on the curve *C* can be expressed as

(5) $$\left\{\begin{array}{l} \alpha_{0C}=\frac{dr}{ds}{|}_{\left(x_{0},y_{0}\right)}≜\dot{r}(x_{0},y_{0})=r_{0C}-r_{-1C}\\ k_{0C}\beta_{0C}=\ddot{r}(x_{0},y_{0})=\dot{\alpha }(x_{0},y_{0})=\alpha_{0C}-\alpha_{-1C} \end{array}\right.$$

where ***r***_0*C*_ is the position vector of point *P*(*x*_0_, *y*_0_) on curve *C* along the direction of *dx/dy*, while ***r***_−1C_ is the position vector of the previous point by *P*(*x*_0_, *y*_0_) on curve *C*. Thus, for the eight-connected neighborhood of the reference point *P*(*x*_0_, *y*_0_), the eight directions by each adjacent pixel correspond to curves *C*_1_ to *C*_8_ on the discrete grayscale surface, whose unit tangent vectors ***α*** and principal normal vectors ***β*** are therefore shown in Figure 5a. At this point, the normal curvature *k_nC_* of curve *C* at point *P*(*x*_0_, *y*_0_) on the discrete grayscale surface Σ can be expressed as the projection of the curvature vector *k_C_**β**_C_* at point *P* onto the normal vector ***n***, namely

(6) $$k_{nC}=k_{C}\beta_{C}\cdot n=k_{C}\cos\theta$$

where ***β****_C_* is the principal normal vector of curve *C* at point *P* and *k_C_* is the curvature of curve *C*, while *θ* is the angle between ***β****_C_* and ***n***. The case of curve *C*_1_ is shown as an example in Figure 5b, and the other seven cases are similar. Normal curvature can serve as a unified indicator for the bending extent of the discrete grayscale surface in the current direction to have distinct geometric significance.

According to the definition of normal curvature above, pixel (*i*, *j*) has eight normal curvatures in eight directions, as shown in Figure 5c. The eight normal curvatures corresponding to the directions of *C*_1_ to *C*_8_, respectively, denoted as *k_n_*_11_ to *k_n_*_33_, are represented as follows:

(7) $$\left\{\begin{array}{l} k_{n11}(i,j)=k_{11}(i,j)\beta_{11}(i,j)\cdot n(i,j)=\frac{\partial^{2}z(i,j)/\partial l_{C1}^{2}}{\left|n_{0}(i,j)\right|}\\ k_{n12}(i,j)=k_{12}(i,j)\beta_{12}(i,j)\cdot n(i,j)=\frac{\partial^{2}z(i,j)/\partial l_{C2}^{2}}{\left|n_{0}(i,j)\right|}\\ k_{n13}(i,j)=k_{13}(i,j)\beta_{13}(i,j)\cdot n(i,j)=\frac{\partial^{2}z(i,j)/\partial l_{C3}^{2}}{\left|n_{0}(i,j)\right|}\\ k_{n21}(i,j)=k_{21}(i,j)\beta_{21}(i,j)\cdot n(i,j)=\frac{\partial^{2}z(i,j)/\partial l_{C4}^{2}}{\left|n_{0}(i,j)\right|}\\ k_{n23}(i,j)=k_{23}(i,j)\beta_{23}(i,j)\cdot n(i,j)=\frac{\partial^{2}z(i,j)/\partial l_{C5}^{2}}{\left|n_{0}(i,j)\right|}\\ k_{n31}(i,j)=k_{31}(i,j)\beta_{31}(i,j)\cdot n(i,j)=\frac{\partial^{2}z(i,j)/\partial l_{C6}^{2}}{\left|n_{0}(i,j)\right|}\\ k_{n32}(i,j)=k_{32}(i,j)\beta_{32}(i,j)\cdot n(i,j)=\frac{\partial^{2}z(i,j)/\partial l_{C7}^{2}}{\left|n_{0}(i,j)\right|}\\ k_{n33}(i,j)=k_{33}(i,j)\beta_{33}(i,j)\cdot n(i,j)=\frac{\partial^{2}z(i,j)/\partial l_{C8}^{2}}{\left|n_{0}(i,j)\right|} \end{array}\right.$$

To summarize, the G^0^, G^1^, and G^2^ continuity parameters are regarded as evaluation indicators for k-order geometric continuity, by which the smoothness of discrete grayscale surfaces is evaluated; and vice versa, the k-order continuity edges of the image can be derived from the k-order discontinuity of the grayscale surface.

### 2.2. Continuity Edges of Grayscale Surface

**Figure 6:**
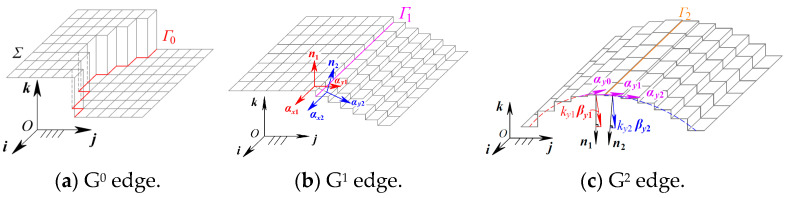
G^k^ Continuity edges.

According to the explanation of the k-order geometric continuity condition and corresponding parameters, the k-order geometric continuity edging discriminate criterion can be expressed as follows: the curve *Γ* is defined as the k-order geometric continuity edge or the *G^k^* (*k* = 0, 1, 2) edge if and only if one side of the curve *Γ* on the image satisfies *G^k^* continuity, while the other side only does not satisfy *G^k^* continuity. Reflecting the zero-/first-/second-order geometric continuity of the surface, the three continuity parameters dominate the three types of continuity edges, as shown in Figure 6.

**Figure 7:**
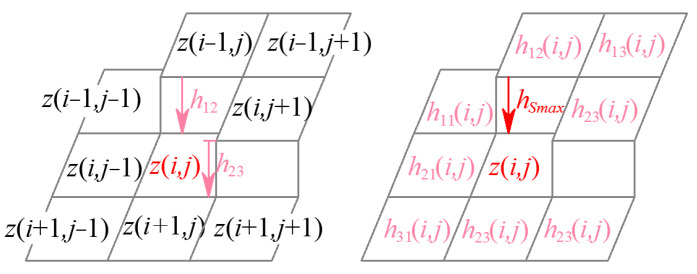
G^0^ edge points and continuity parameters.

Dominated by the G^0^ continuity parameters, the G^0^ edge is defined by the G^0^ continuity condition of the grayscale surface. If the position (namely the function value) of the grayscale surface Σ is discontinuous along the curve *Γ*_0_, the curve *Γ*_0_ is defined as a G^0^ edge of the grayscale surface, as shown in Figure 6a. The discriminate condition for the G^0^ edge is presented as follows: except for the boundary points of the image, one pixel is determined as a G^0^ edging point if the grayscale gradients by pixels in the eight-connected neighborhood satisfy

(8) $$\left\{\begin{array}{l} \exists h_{s}\in \{h_{11},h_{12},h_{13},h_{21},h_{23},h_{31},h_{32},h_{33}\},h_{s}\geq S_{h\max}\\ \exists h_{s}\in \left\{\left|h_{11}\right|,\left|h_{12}\right|,\left|h_{13}\right|,\left|h_{21}\right|,\left|h_{23}\right|,\left|h_{31}\right|,\left|h_{32}\right|,\left|h_{33}\right|\right\},h_{s}\leq S_{h\min} \end{array}\right.$$

where *h*_11_ to *h*_33_ are the G^0^ continuity parameters, namely the grayscale gradients of pixels in the eight-connected region for (*i*, *j*), as shown in Figure 7. When at least one of the eight grayscale gradients is greater than the specified threshold *S_hmax_*, the pixel (*i*, *j*) is determined to be a G^0^ edge point of the grayscale image. The second equation of (9) is the continuity equation for G^0^ edge points to ensure the continuity of edges and eliminate the isolated edge points. Thus, pixel points satisfying the G^0^ edge discriminate criterion in Equation (8), except the boundary points of the image are defined as G^0^ edge points of the grayscale image to compose a G^0^ edging image.

**Figure 8:**
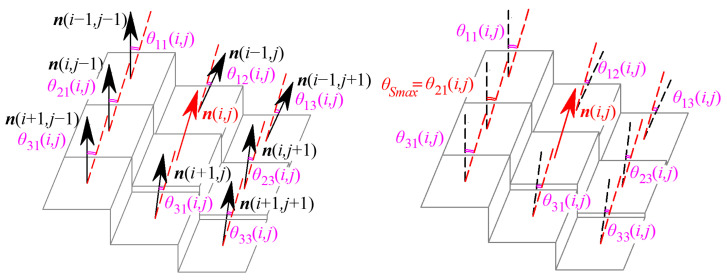
G^1^ edge points and continuity parameters.

Similarly, the G^1^ edge is dominated by the G^1^ continuity parameters and defined by the G^1^ continuity condition of the grayscale surface. If the normal vector direction of the grayscale surface Σ is discontinuous along the curve *Γ*_1_, the curve *Γ*_1_ is defined as a G^1^ edge of the grayscale surface, as shown in Figure 6b. The discriminate condition for the G^1^ edge is presented as follows: except for the boundary points of the image, one pixel is determined as a G^1^ edging point if the included vector angle by pixels in the eight-connected neighborhood satisfies

(9) $$\left\{\begin{array}{l} \theta_{11}(i,j)=\arccos(n(i,j)\cdot n(i-1,j-1))\\ \theta_{12}(i,j)=\arccos(n(i,j)\cdot n(i-1,j))\\ \theta_{13}(i,j)=\arccos(n(i,j)\cdot n(i-1,j+1))\\ \theta_{21}(i,j)=\arccos(n(i,j)\cdot n(i,j-1))\\ \theta_{23}(i,j)=\arccos(n(i,j)\cdot n(i,j+1))\\ \theta_{31}(i,j)=\arccos(n(i,j)\cdot n(i+1,j-1))\\ \theta_{32}(i,j)=\arccos(n(i,j)\cdot n(i+1,j))\\ \theta_{33}(i,j)=\arccos(n(i,j)\cdot n(i+1,j+1))\\ \exists \theta_{s}\in \{\theta_{11},\theta_{12},\theta_{13},\theta_{21},\theta_{23},\theta_{31},\theta_{32},\theta_{33}\},\theta_{s}\geq S_{n\max}\\ \exists \theta_{s}\in \left\{\left|\theta_{11}\right|,\left|\theta_{12}\right|,\left|\theta_{13}\right|,\left|\theta_{21}\right|,\left|\theta_{23}\right|,\left|\theta_{31}\right|,\left|\theta_{32}\right|,\left|\theta_{33}\right|\right\},\theta_{s}\leq S_{n\min} \end{array}\right.$$

where *θ*_11_ to *θ*_33_ are the included angles of normal vectors for the pixel (*i*, *j*) by pixels in the eight-connected region, which are directly determined by the G^1^ continuity parameters, as is shown in Figure 8. When at least one of the eight included angles is greater than the specified threshold *S_nmax_*, the pixel (*i*, *j*) is determined to be the G^1^ edge point of the grayscale image. The last equation of Equation (10) is the continuity equation for G^1^ edge points to ensure the continuity of edges and eliminate the isolated edge points. Thus, pixel points satisfy the G^1^ edge discriminate criterion in Equation (9), except the boundary points of the image are defined as G^1^ edge points of the grayscale image to compose a G^1^ edging image.

**Figure 9:**
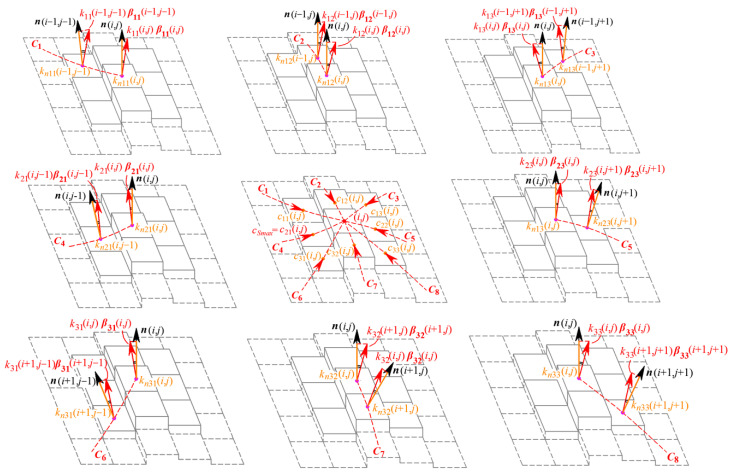
G^2^ edge points and continuity parameters along eight directions.

Similar to the G^0^ and G^1^ edges, the G^2^ edge is dominated by the G^2^ continuity parameters and defined by the G^2^ continuity condition of the grayscale surface. If the normal curvature of the grayscale surface Σ is discontinuous along the curve *Γ*_2_ in any direction, the curve *Γ*_2_ is defined as a G^2^ edge of the grayscale surface, as is shown in Figure 6c. The discriminate condition for the G^2^ edge is presented as follows: except for the boundary points of the image, one pixel is determined as a G^2^ edging point if the normal curvature gradients by pixels in the eight-connected neighborhood satisfy

(10) $$\left\{\begin{array}{l} c_{11}(i,j)=k_{n11}(i,j)-k_{n11}(i-1,j-1)\\ c_{12}(i,j)=k_{n12}(i,j)-k_{n12}(i-1,j)\\ c_{13}(i,j)=k_{n13}(i,j)-k_{n13}(i-1,j+1)\\ c_{21}(i,j)=k_{n21}(i,j)-k_{n21}(i,j-1)\\ c_{23}(i,j)=k_{n23}(i,j)-k_{n23}(i,j+1)\\ c_{31}(i,j)=k_{n31}(i,j)-k_{n31}(i+1,j-1)\\ c_{32}(i,j)=k_{n32}(i,j)-k_{n32}(i+1,j)\\ c_{33}(i,j)=k_{n33}(i,j)-k_{n33}(i+1,j+1)\\ \exists c_{s}\in \{c_{11},c_{12},c_{13},c_{21},c_{23},c_{31},c_{32},c_{33}\},c_{s}\geq S_{k\max}\\ \exists c_{s}\in \left\{\left|c_{11}\right|,\left|c_{12}\right|,\left|c_{13}\right|,\left|c_{21}\right|,\left|c_{23}\right|,\left|c_{31}\right|,\left|c_{32}\right|,\left|c_{33}\right|\right\},c_{s}\leq S_{k\min} \end{array}\right.$$

where *k_n_*_11_ to *k_n_*_33_ are the G^2^ continuity parameters, which are the normal curvatures for the pixel (*i*, *j*) in eight directions; *c*_11_ to *c*_33_ are the normal curvature gradients for the pixel (*i*.*j*) by each pixel in the eight-connected region along the corresponding eight directions, respectively shown in Figure 9. When at least one of the eight normal curvature gradients is greater than the specified threshold *S_kmax_*, the pixel (*i*, *j*) is determined to be the G^2^ edge point of the grayscale image. The last equation of (11) is the continuity equation for G^2^ edge points to ensure the continuity of edges and eliminate the isolated edge points. Thus, pixel points satisfying the G^2^ edge discriminate criterion in (10), except the boundary points of the image are defined as G^2^ edge points of the grayscale image to compose a G^2^ edging image.

### 2.3. Feature Edges of the Grayscale Surface

**Figure 10:**
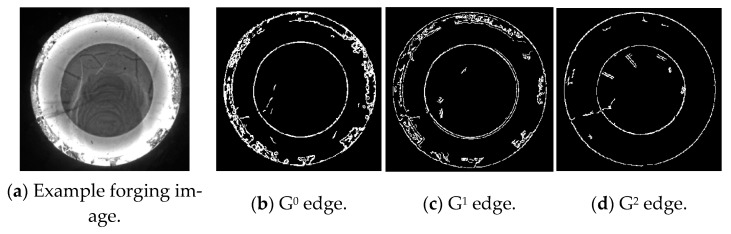
Continuity edges for an example grayscale forging image.

The continuity edges have been provided above based on the continuity parameters, by which an example forging image, as well as the corresponding continuity edgings, are shown in Figure 10. The three types of edging images indicate that the G^0^ edging reflects the most prominent geometric features and textures on the surface of the forging part, while the grayscale information by the G^1^ and G^2^ edges is relatively weak, corresponding to the minor textures on the forging surface and the boundary of the grayscale transition areas outside the forging part. The characteristics between the G^0^ edging and G^1^ and G^2^ edges are discussed below, by which feature edges are defined.

In the passive visual measurement process of hot forgings, the outermost edge of the forging part needs to be extracted for image segmentation. Thus, the outer edge serves as the reference for feature extraction, matching, and reconstruction, which is closely related to measurement accuracy. As mentioned, the continuity edge proposed in this paper contains forging contour information; thus, it is a simple and feasible method to extract the outer edge based on the continuity edge directly. Therefore, the outermost continuity edges are referred to as the continuity feature edges in this paper, abbreviated as feature edges. Three types of feature edges can be extracted separately from the continuity edges, namely the G^0^/G^1^/G^2^ feature edges.

**Figure 11:**
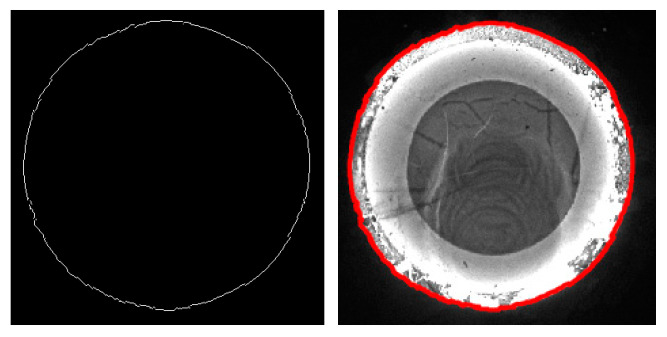
G^0^ feature edge and its segmentation performance.

**Figure 12:**
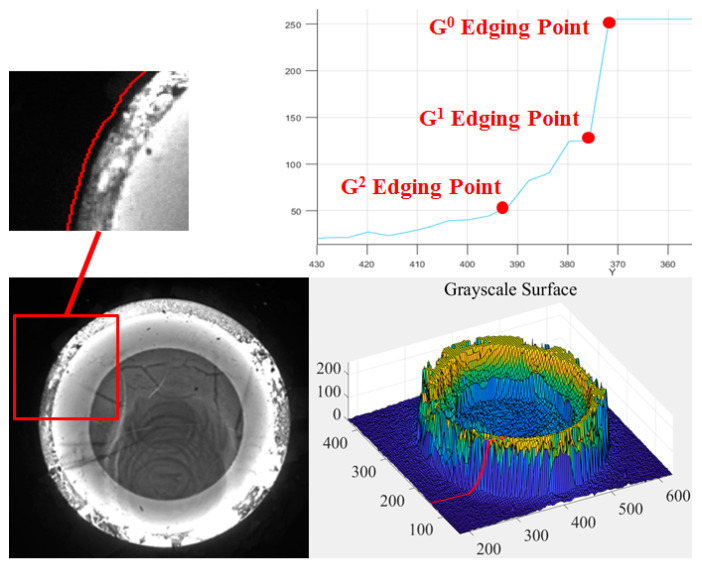
Grayscale distribution characteristics of the continuity feature edges.

**Figure 13:**
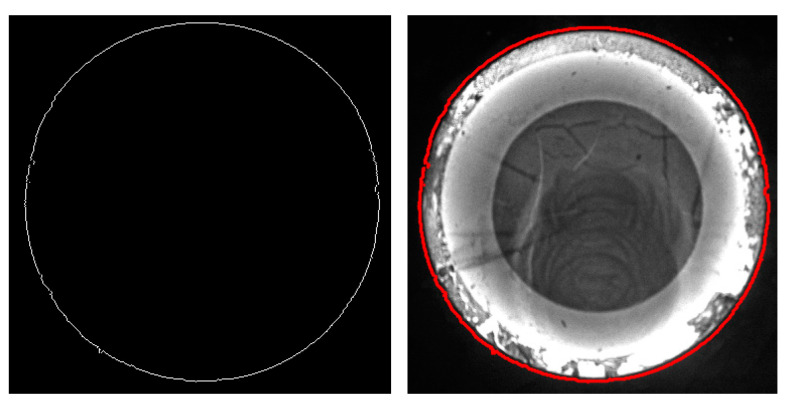
G^2^ feature edge and its segmentation performance.

Among the three types of feature edges, the G^0^ feature edging is most likely to correspond to the target outer contour for hot forgings since it contains the most distinct grayscale characteristics for G^0^ edges. Admittedly, the required outermost edge can be extracted by G^0^ feature edging; however, the oxide scales on the forging surface are likely to cause low grayscale textures in the image, which may lead to poor contour smoothness and low confidence outliers and thus poor measurement accuracy, as shown in Figure 11. On the contrary, the G^1^/G^2^ feature edges are provided with weaker grayscale characteristics, so the G^1^ and G^2^ feature edges are actually located in the grayscale transition area outside the forging contour in the background, where the grayscale changes less distinctly. In other words, the area segmented by the G^1^/G^2^ feature edges contains part of the background illuminated by thermal radiation, so as to be slightly larger than the forging part. Interestingly, this grayscale distribution characteristic coincides with the situation of gradual edges described in [44] for fastener images. The grayscale distribution characteristics of the continuity feature edges are shown in Figure 12. The target area enveloped by G^2^ feature edges is prone to being larger and fully containing the target forging part, which makes the G^2^ feature edge available to be the initial control point for the Snake model [1], as shown in Figure 13. The GC Snakes model proposed later in this paper will also utilize this principle of the G^2^ feature edges.

## 3. The Proposed Geometric Continuity Snakes Model

The continuity parameters, derived continuity edges, and corresponding feature edges have been proposed in the previous section. In this section, a new Snakes image force, together with an external energy functional, is proposed based on the continuity parameters of grayscale surfaces, by which a geometric continuity active contour model, namely the GC Snakes model, is established. On this basis, an effective and robust image segmentation method is presented by combining the GC Snakes model and G^2^ feature edges for forging images.

### 3.1. Basic Principles and Deficiency of the Snakes

The classic Snakes model was developed by Kass. M et al. [33] and is a closed parameterized curve defined near the target boundary. The active contour curve is called the energy curve, which moves towards the target boundary continuously under the influence of external and internal energy. When the energy of the curve reaches its minimum, the corresponding curve becomes the target segmentation boundary. The initial contour curve of the Snakes model can be represented as a closed parametric curve ***v***(*s*) = [*x*(*s*), *y*(*s*)] near the target boundary, where *s* ∈ [0, 1] and is the arc length parameter. If represented by *C_S_*, the control point set *C_S_* of the Snakes model needs to satisfy the contour curve, that is

(11) $$C_{S}:v(s)=\left[x(s),y(s)\right],s\in \left[0,1\right]$$

Composed of internal and external energy, the energy functional of the Snakes active contour can be represented as

(12) $$\left\{\begin{array}{l} E_{Snake}(v)=E_{int}(v)+E_{ext}(v)=\int_{s}\left(E_{int}(v(s))+E_{ext}(v(s))\right)\;ds\\ E_{int}(v)=\frac{1}{2}\left[\alpha (s){\left|\frac{\partial }{\partial s}v(s)\right|}^{2}+\beta (s){\left|\frac{\partial^{2}}{\partial s^{2}}v(s)\right|}^{2}\right] \end{array}\right.$$

where *E_int_* represents the internal energy, including elastic and bending energy, which are used to control the elastic deformation of the contour line. *E_ext_* represents the internal energy and is responsible for driving the curve to converge toward the boundaries of the image. Determined by image features, the external energy can be represented by local features at the positions of control points or on their connecting lines, such as

(13) $$E_{ext}(v(s))=-{\left|▽I(x,y)\right|}^{2}$$

or

(14) $$E_{ext}(v(s))=-{\left|▽\left[G_{\sigma }(x,y)*I(x,y)\right]\right|}^{2}=-{\left|▽I_{G}(x,y)\right|}^{2}$$

**Figure 14:**
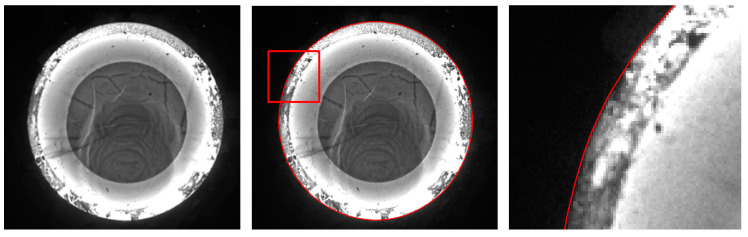
Segmentation performance and detailed view for forging image with high thermal radiation by the Snakes.

**Figure 15:**
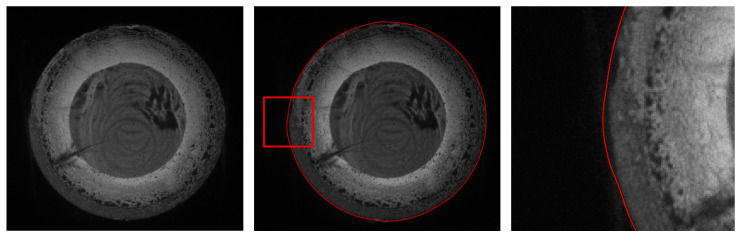
Segmentation performance and the unsatisfying detail for forging image with low thermal radiation by the Snakes.

According to the variational principle, the energy functional of the Snakes model can be minimized to achieve the segmentation of grayscale images for hot forgings. However, there exist several limitations in real-time forging image processing for the Snakes. Firstly, manually selected control points around the target part to be segmented are generally required as the starting position of the active contour for Snakes, based on which the energy functional is iteratively solved to achieve target image segmentation. The manual selection of control points can be relatively complex, inefficient, and unstable, making it unsuitable for the real-time segmentation of grayscale images in the online measurement process of forging parts. Secondly, the image force of the Snakes model is entirely determined by the simple calculation of image gradients in two directions, according to Equations (13) and (14). The Snakes image force will work in areas with relatively large local grayscale gradients and obvious grayscale features; however, the image force is relatively weak for cases where the grayscale features are not significantly notable between target and background, which causes quite weak convergence to the target contour for the active contour, as shown in Figure 14 and Figure 15.

Figure 14 shows the segmentation results of a high-temperature forging image using the Snakes model. Having higher thermal radiation intensity, the area of a high-temperature forging has higher grayscale in the image under the same acquisition conditions. Therefore, the grayscale characteristics of the parts to be segmented are clearly distinguished from the background area, so the Snakes can provide good segmentation results. But for areas with more surface textures formed by oxide scale, the surface temperature is prone to cooling down, which results in lower surface temperature, leading to the lower grayscale in the image at the forging boundary. In this case, the grayscale gradient between the hot forging and the background is non-significant, so the image force is relatively weak, causing poor segmentation performance in the local area. On the other hand, Figure 15 shows the segmentation results of a low-temperature hot forging image using the Snakes model. The lower thermal radiation intensity results in low grayscale, as well as weaker grayscale characteristics in the image. Thus, the image force near the active contour can be relatively weak, generating poor convergence accuracy of the active contour, which makes it hard to meet the measurement accuracy.

In response to the limitations above, a new external force and energy functional, as well as a generating method for the initial control points, is proposed below based on the grayscale surface continuity to form a new geometric continuity active contour model, namely the GC Snakes model.

### 3.2. GC Snakes

The previous section briefly introduced the basic principles and main limitations of the Snakes. In this section, an image segmentation algorithm suitable for real-time processing of hot forging images, namely the GC Snakes, is proposed based on the geometric properties of the grayscale surface.

Regarding the limitation of requesting manually selected control points for the Snakes, an effective determination method for the initial control point set is proposed here. As is analyzed in Section 2.3, the area enveloped by the G^2^ feature edge is slightly larger than the target and thus can fully encompass the target forging part, which is exactly consistent with the properties of the initial control points for the Snakes. Therefore, it has wide applicability in forging image processing to detect the second-order geometric continuity edge and then extract the corresponding feature edge to form initial control points. If *C_GS_* represents the initial control points of the GC Snakes and *C_G_* represents the G^2^ feature edge point set, the control points *C_GS_* are supposed to satisfy

(15) $$C_{GS}\subset C_{G},C_{GS}:v_{GS}=[x_{GS},y_{GS}],\;x_{GS}={\left[\begin{array}{c} x_{0}\\ x_{1}\\ x_{2}\\ \ldots \\ x_{N-2}\\ x_{N-1} \end{array}\right]}_{N\times 1},\;y_{GS}={\left[\begin{array}{c} y_{0}\\ y_{1}\\ y_{2}\\ \ldots \\ y_{N-2}\\ y_{N-1} \end{array}\right]}_{N\times 1}$$

so that the initial control point set *C_GS_* of the active contour can be determined by the G^2^ feature edging point set or its subset by reducing sampling.

For the classic Snakes, the image force and external energy are determined by the image gradients in two directions, expressed in the two forms in Equations (13) and (14). If *g_x_* and *g_y_* represent the grayscale gradients along the *x* and *y* directions at pixel point (*x*, *y*), the external energy of the classic Snakes can be represented as

(16) $$\left\{\begin{array}{l} E_{ext}(v)=-{\left|▽I(x,y)\right|}^{2}=-{\left[g_{x}{\left(x,y\right)}^{2}+g_{y}{\left(x,y\right)}^{2}\right]}^{2}\\ E_{ext}(v)=-{\left|▽I_{G}(x,y)\right|}^{2}=-{\left[g_{Gx}{\left(x,y\right)}^{2}+g_{Gy}{\left(x,y\right)}^{2}\right]}^{2} \end{array}\right.$$

If the geometric properties of the grayscale surface *h*(*x*, *y*, *z*) = 0 for the forging image are taken into account, and based on the definition of G^0^ continuity parameters proposed in Section 2.1, Equation (16) can be rewritten as

(17) $$E_{ext}(v)=-{\left[h_{12}{\left(x,y\right)}^{2}+h_{21}{\left(x,y\right)}^{2}\right]}^{2}$$

It can be seen that the external energy functional of the Snakes is only defined by two unidirectional gradients, which can hardly reflect the local grayscale properties of the image, so the image force of the Snakes is easily affected by the grayscale of local discrete pixels, that is, greatly prone to being affected by the local grayscale changes near the target area to be segmented; in terms of areas with non-distinct grayscale differentiation from the background, it is more difficult to reflect the changes in grayscale distribution by using two directional gradients. Therefore, the classic Snakes model has poor segmentation performance for forging parts with low grayscale, which has been shown in Figure 15.

To solve the problem of poor segmentation stability for the classic Snakes, this paper defines an external energy functional for GC Snakes using continuity parameters from the geometric properties of grayscale surfaces. Based on the definition and characteristics of the three types of feature parameters, the G^0^ feature parameters are most direct to reflect the grayscale distribution characteristics of the forging image. This property can be most intuitively reflected through the unidirectional distribution curve on the grayscale surface, shown in Figure 12, where the G^0^ feature edge points precisely correspond to the target contour for the part to be segmented. Therefore, it has a significant theoretical basis to realize precise convergence to the target contour by introducing the zero-order geometric feature parameters into external energy and force. Thus, for a given grayscale image *I* of a hot forging, the GC Snakes’ external energy can be defined as

(18) $$E_{GCext}(v)=-{\left[\sum_{i=1}^{8}h_{gi}{\left(x,y\right)}^{2}\right]}^{2}$$

where *h_gi_* (*x*, *y*) are the eight-directional external energy parameters of GC Snakes at the reference pixel (*x*, *y*) in the image *I_g_*. And *G_σ_* (*x*, *y*) represents a two-dimensional Gaussian kernel with a standard deviation of *σ*, so *I_g_* and *h_gi_* satisfy

(19) $$\left\{\begin{array}{l} I_{g}(x,y)=G_{\sigma }(x,y)*I(x,y)\\ h_{g1}(x,y)=h_{g11}(x,y),\;h_{g2}(x,y)=h_{g12}(x,y),\;h_{g3}(x,y)=h_{g13}(x,y)\\ h_{g4}(x,y)=h_{g21}(x,y),\;h_{g5}(x,y)=h_{g23}(x,y)\\ h_{g6}(x,y)=h_{g31}(x,y),\;h_{g7}(x,y)=h_{g32}(x,y),\;h_{g8}(x,y)=h_{g33}(x,y) \end{array}\right.$$

**Figure 16:**
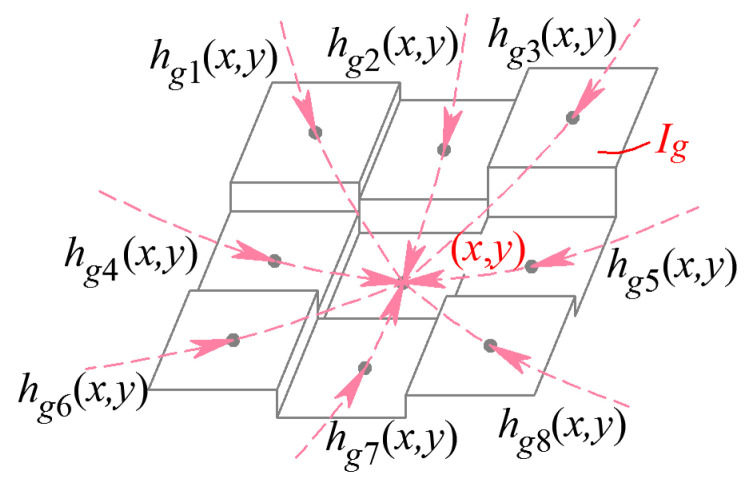
External energy parameters of GC Snakes.

The external energy parameters of GC Snakes are shown in Figure 16. Based on the defined external energy, the energy functional of the GC Snakes active contour curve can be expressed as

(20) $$E_{GCSnake}(v)=E_{int}(v)+E_{GCext}(v)=\int_{s}\left(E_{int}(v(s))+E_{GCext}(v(s))\right)\;ds$$

where *E_int_* is the internal energy, which can be represented by referring to Section 3.1. *E_GCext_* is the defined geometric continuity external energy that drives the active contour curve to converge toward the target contour.

According to the variational principle, the necessary condition for minimizing the energy functional of *E_GCsnake_* is that the active contour ***v***(*s*) satisfies the following Euler-Lagrange equation:

(21) $$\alpha v^{\prime \prime }(s)-\beta v^{\left(4\right)}(s)-▽E_{GCext}=0$$

which can also be rewritten as

(22) $$F_{int}(v)+F_{GCext}(v)=0$$

where the internal force *F_int_* corresponds to internal energy and controls the stretching and bending of the spline curve, while the external force *F_GCext_* corresponds to external energy *E_GCext_* to drive the active contour to move toward the target contour defined by geometric continuity parameters. When solving Equation (22), a time parameter *t* is introduced to treat the curve ***v***(*s*) as a function of ***v***(*s*, *t*) that changes over time, so the image segmentation process can be transformed into solving the following partial differential equation as

(23) $$\frac{\partial v}{\partial t}=\alpha v^{\prime \prime }(s,t)-\beta v^{\left(4\right)}(s,t)-▽E_{GCext}$$

**Figure 17:**
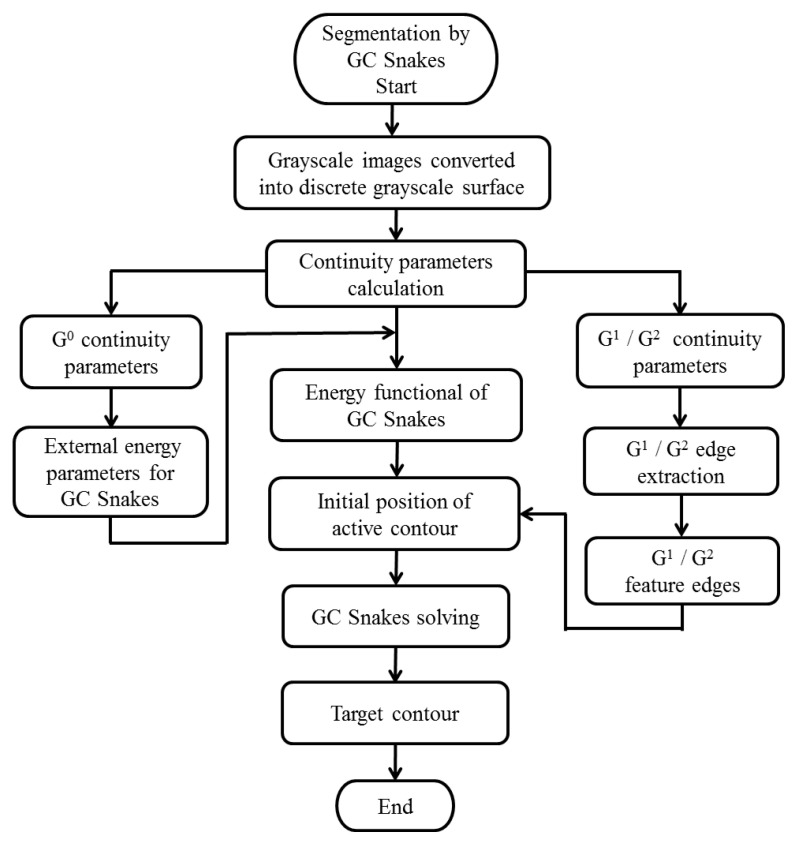
The flowchart for GC Snakes.

During the solving process, curve ***v***(*s*) moves continuously by the internal and external forces. When the active contour ***v***(*s*) is stable, the value on the right side of Equation (23) is taken as 0. At this point, the active contour ***v***(*s*) is the desired target contour. Therefore, the implementation process for the algorithm of GC Snakes is shown by a flow chart in Figure 17.

### 3.3. Image Segmentation by GC Snakes

In this section, the proposed GC Snakes is validated for image segmentation and then compared with the segmentation method described in [1] and GVF Snakes. To validate the segmentation performance for grayscale forging images under different working conditions, the performance and efficiency are verified by grayscale images in real-time measuring of three types of hot forgings as large-sized forging parts with high and low temperatures, and a small-sized forging part, respectively. To simulate a real online measurement environment, the images are collected in the same acquisition conditions, namely all passive visual grayscale images collected by a 905 nm narrowband filter.

**Figure 18:**
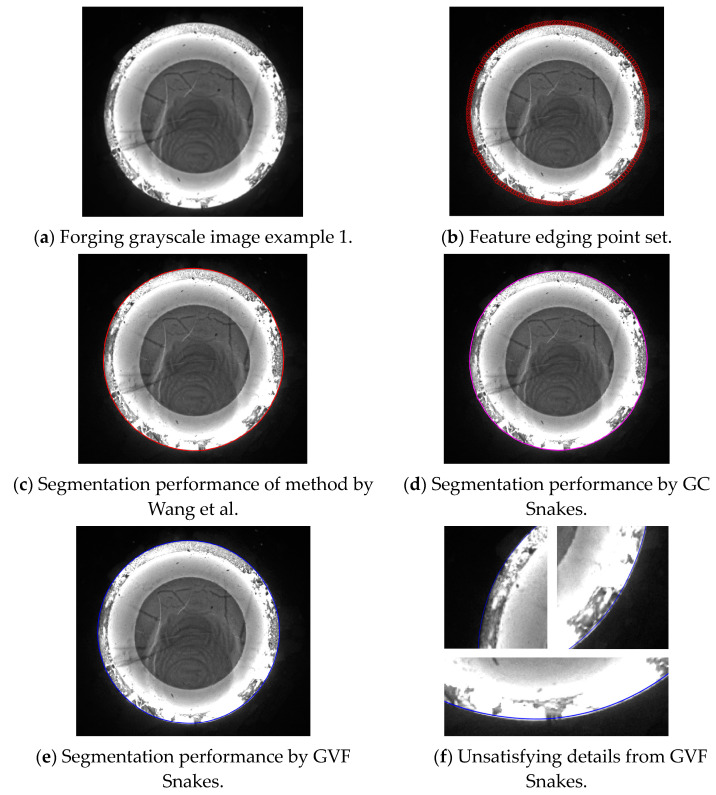
Segmentation performance for forging image with large dimensions and high thermal radiation [1].

**Figure 19:**
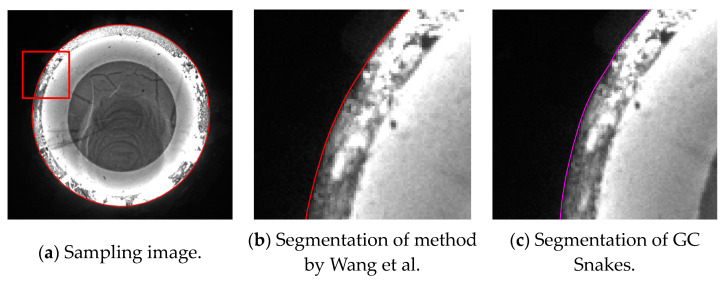
Detailed view for segmentation of forging image with large dimensions and high thermal radiation [1].

The first experiment validates the segmentation performance for GC Snakes by an example image of forging with large dimensions and high thermal radiation. By performing geometric continuity edge detection on the grayscale image of the high-temperature forging shown in Figure 18a, the G^2^ feature edge can be extracted. The subset of feature edge points *C_GS_* from reduced sampling is determined to be the initial control points for the GC Snakes active contour, as shown in Figure 18b. Substituting the coordinates of control points into GC Snakes, the target contour of the forging part to be tested can be calculated using the GC Snakes active contour method. The image segmentation performance of a large-sized high-temperature hot forging is shown in Figure 18d; as a comparison, Figure 18c shows the segmentation result of the method described in [1], while Figure 18e shows the segmentation result of GVF Snakes, whose local accuracy can be unsatisfying, with a detailed view shown in Figure 18f. According to the experimental results, GC Snakes can extract the target boundaries of the tested forging part with better accuracy and continuity compared to the method in [1] and GVF Snakes, which can meet the image segmentation requirements of forging measurement; in terms of the areas where there is much oxide scale at the boundary, resulting in non-significant grayscale gradient difference between the forging and background, shown in Figure 19, the segmentation method by GC Snakes performs much better than the feature-edge-based Snakes segmentation method described in [1], with the target contour fitting the forging contour better. The image force defined by GC Snakes has satisfactory stability and robustness for areas with different grayscale gradient variations. Therefore, it has also ideal performance for areas with unremarkable gradients between the target and background, which ensures reliable convergence to the target boundary for the active contour. In addition, the segmentation efficiency of GC Snakes is also satisfying, as the calculational times are 4.75 s for GC Snakes, 4.67 s for GVF Snakes, and 10.92 s for the method in [1] on a normal PC (Intel Core i5-8400 CPU, 16 G RAM, Manufacturer: Microsoft China, sourced in Dalian, China). As a result, GC Snakes has been a more satisfactory method for the real-time segmentation of forging images in view of its performance and efficiency.

**Figure 20:**
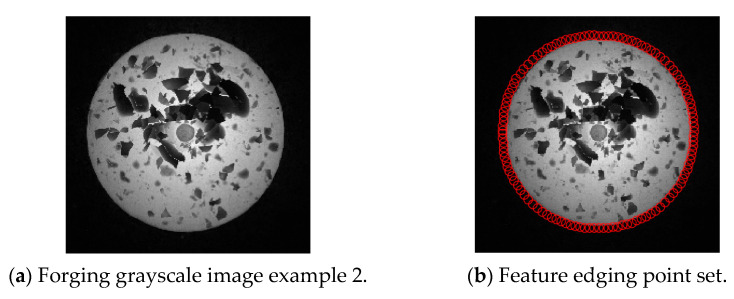

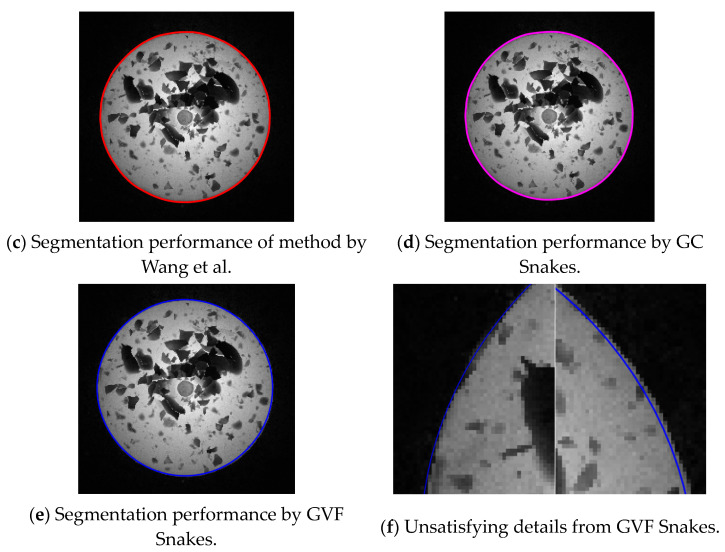
Segmentation performance for forging image with small dimension [1].

**Figure 21:**
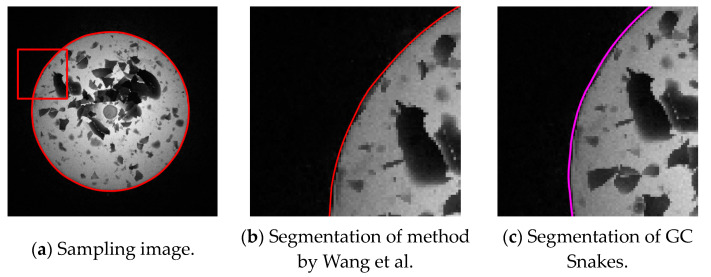
Detailed view for segmentation of forging image with small dimension [1].

The second experiment validates the segmentation performance for GC Snakes by an example image of forging with small dimensions. Based on the continuity edge detection method, the G^2^ edge, and the feature edge are extracted for the grayscale image of the small dimension forging shown in Figure 20a, by which the subset of feature edge points *C_GS_* is established as initial control points for the GC Snakes active contour, as shown in Figure 20b. Substituting the coordinates of control points into GC Snakes, the target contour of the forging part can be calculated using the GC Snakes active contour method. The image segmentation performance of a small-sized hot forging is shown in Figure 20d; as a comparison, Figure 20c shows the segmentation result of the method described in [1], while Figure 20e shows the segmentation result of GVF Snakes, whose local accuracy can be also unsatisfying, with a detailed view shown in Figure 20f. In accordance with the experimental results, GC Snakes comparably extracts the target boundaries of the forging part with better accuracy and continuity compared to the method in [1] and GVF Snakes, which can meet the image segmentation requirements of forging measurement; on the other hand, due to the relatively uniform distribution of thermal radiation on the surface of small-sized forgings, the situation in which the forging and background are not significantly distinguished in Experiment 1 is relatively not obvious. However, there are still local areas in which non-significant grayscale gradient difference occurs between the forging and background, where GC Snakes also shows ideal segmentation performance. The image force and external energy defined in the GC Snakes segmentation method ensure good stability and robustness, as shown in Figure 21. In addition, the segmentation efficiency of GC Snakes is also satisfying, as the calculational times are 3.08 s for GC Snakes, 4.52 s for GVF Snakes, and 4.61 s for the method in [1]. As a result, GC Snakes has been a more satisfactory method for the real-time segmentation of forging images in view of its performance and efficiency.

**Figure 22:**
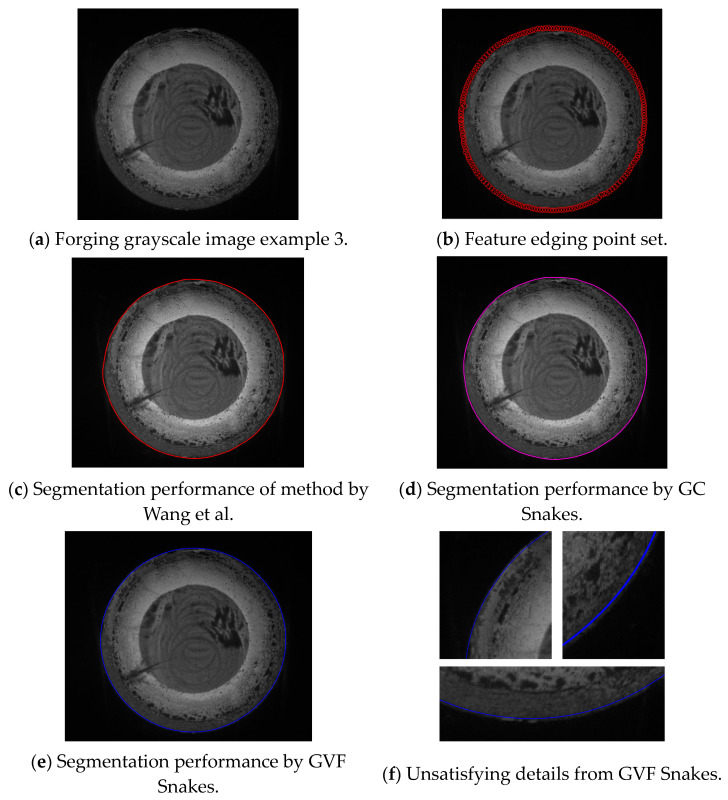
Segmentation performance for forging image with large dimension and low thermal radiation [1].

**Figure 23:**
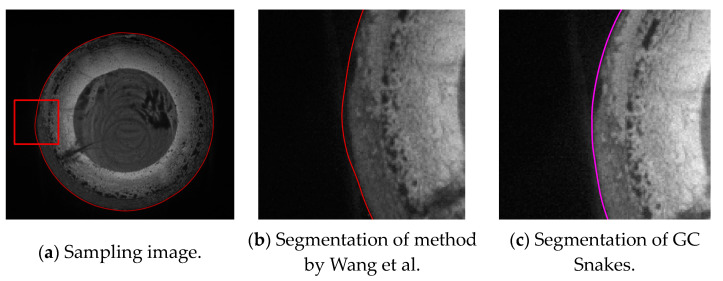
Detailed view for segmentation of forging image with large dimensions and low thermal radiation [1].

The third experiment validates the segmentation performance for GC Snakes by an example image of forging with large dimensions and low thermal radiation. Similarly, geometric continuity edge detection is performed on the grayscale image of the low-temperature forging shown in Figure 22a, based on which the feature edging point set *C_GS_* can be extracted as initial control points for GC Snakes, as shown in Figure 22b. By substituting the coordinates of control points into the GC Snakes, the target contour of the forging part to be tested can be calculated. The image segmentation performance of a large-sized low-temperature hot forging is shown in Figure 22d; as a comparison, Figure 22c shows the segmentation result by the method described in [1], while Figure 22e shows the segmentation result by GVF Snakes, whose local accuracy can be unsatisfying, with a detailed view shown in Figure 22f. According to the experimental results, for situations where the grayscale gradient of the part is rather weak between the forging and background, causing much segmentation difficulty, GC Snakes can still extract the target boundary with better accuracy and continuity compared to the method in [1] and GVF Snakes, which can meet the image segmentation requirements for forging measurement. Due to the unclear boundary between the forging and the background, the segmentation performance of the method described in [1] is unsatisfying, with the contour hardly converging to the target position; in contrast, the image force defined by the GC Snakes model still has clear theoretical significance even when the gradient changes are not significant, as shown in Figure 23. In addition, the segmentation efficiency of GC Snakes is also satisfying, as the calculational times are 6.82 s for GC Snakes, 5.68 s for GVF Snakes, and 9.54 s for the method in [1]. As a result, GC Snakes has been a more satisfactory method for the real-time segmentation of forging images in view of its performance and efficiency. Therefore, GC Snakes has been proven to have stable convergence, robust performance, and satisfying efficiency for images of various grayscale gradient distributions, and it should be adaptable for various temperatures and thermal radiation intensities of hot forgings under real online measurement.

## 4. Measurement Experiments for Hot Forging Based on GC Snakes

The above sections have proposed the GC Snakes model and the corresponding image segmentation approach for hot forgings, and the experiments have been developed as well to verify the segmentation performance and robustness for forging images with different grayscale distributions. In this section, experimental schemes of geometric parameter measurement for hot forgings are provided, by which testing equipment is built to conduct verification experiments. The measuring results are to be compared with the method described in [1] to further verify the superiority of GC Snakes.

### 4.1. Experimental Schemes of Geometric Parameter Measurements for Hot Forging

As mentioned, image segmentation has a significant impact on the measurement results with the generated boundary, serving as the reference for feature extraction. Thus, segmentation results are verified by measuring the dimension and positioning for a forging test part, where the segmented contour is regarded as the boundary for feature extraction.

**Figure 24:**
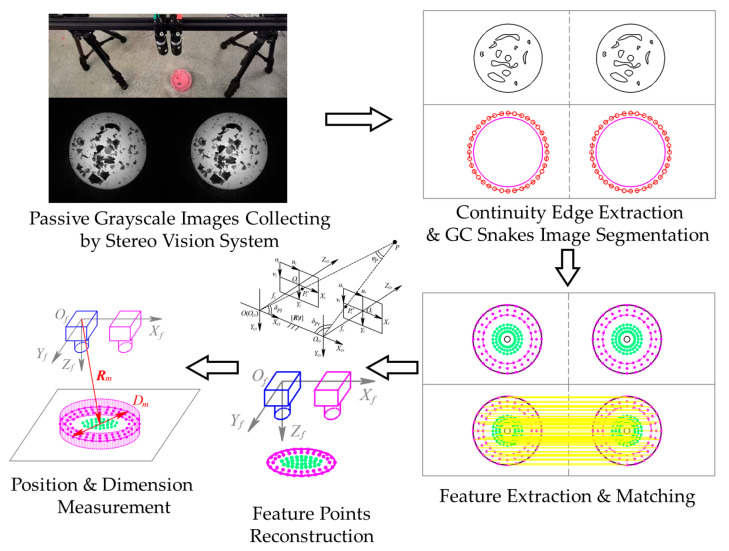
The experimental scheme for the geometric parameter measurement.

Simulating the real-time measuring for hot forging, passive visual measurement is equally adopted in the validation for GC Snakes in the measurement of the forging test part, where narrow band filters are used to collect the grayscale images to compose binocular vision measurement to realize dimension and positioning measuring for the forgings. During the measurement process, grayscale forging images are captured from binocular cameras and then extracted for feature edges, after which the images are segmented by GC Snakes; hence, within the region surrounded by the target contour, feature points can be extracted, matched, and then reconstructed in the measurement coordinate system. Containing feature points on the upper surface as well as adjacent to the outer contour, the reconstructed features reflect the geometric parameters of the forging test part well, the most essential of which are the dimension and the position, determined by the enveloped cylinder for the feature points. The experimental scheme for the dimension and positioning measurement process of the hot forgings is shown in Figure 24.

**Figure 25:**
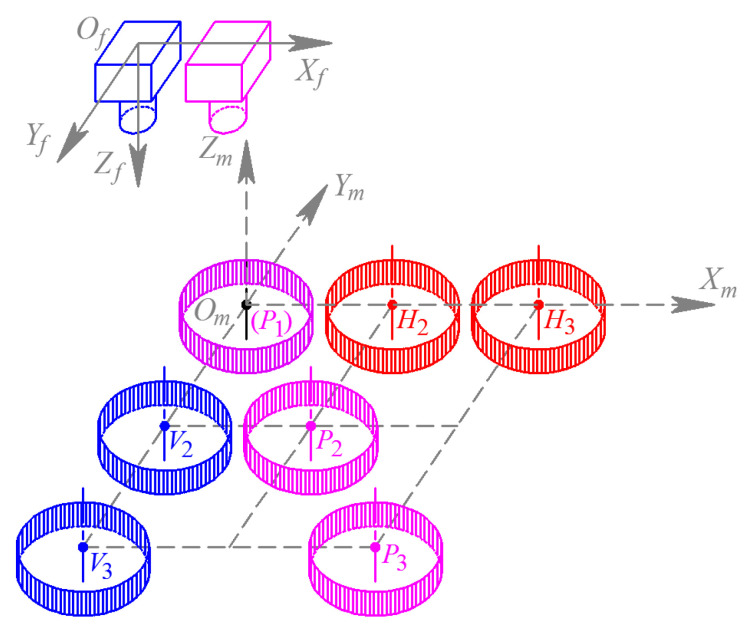
The test plan for the validation measurements.

According to the proposed experimental scheme of the dimension and positioning measurement for hot forgings, the test plan for validation of GC Snakes is shown in Figure 25. Establishing a measurement coordinate system {*O_m_*; *X_m_*, *Y_m_*, *Z_m_*} at the initial measurement position of the forging, three measurement positions in the directions of axis *X_m_* and *Y_m_* and a general direction are set up, respectively, where ***X****_m_* = [0, 30, 60]*^T^* and ***Y****_m_* = [0, 30, 60]*^T^*; thus, nine measurement positions are established to operate geometric parameters measurements for validation experiments. The dimensions and positioning of the forging test part are measured and compared with those by the image segmentation method described in [1], so the performance and stability of the GC Snakes is verified.

### 4.2. Geometric Parameter Measurement by GC Snakes

**Figure 26:**
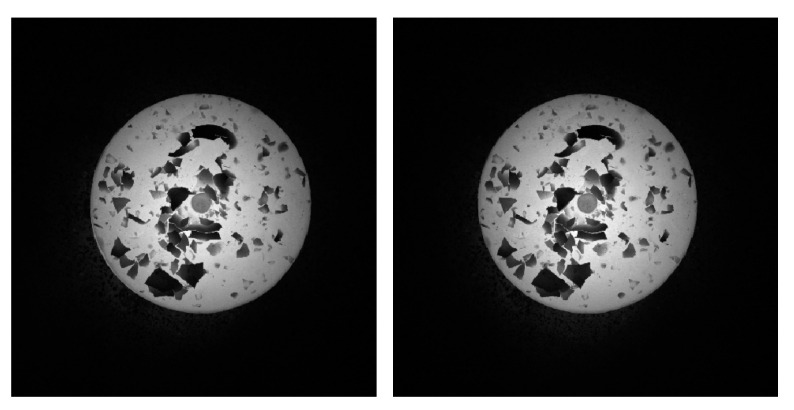
Grayscale stereo vision images of the testing forging part.

**Figure 27:**
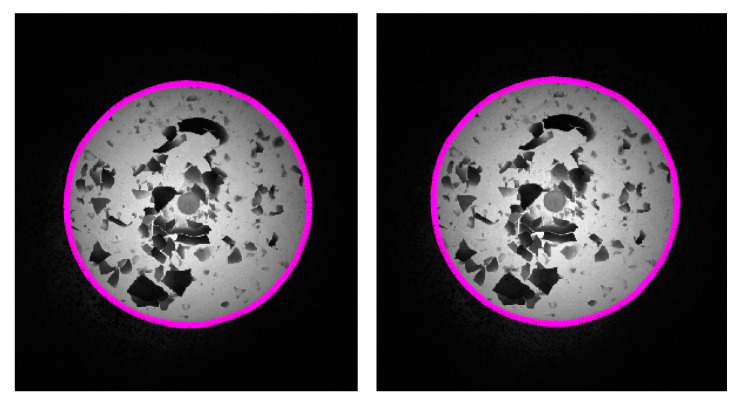
Segmentation performance for the stereo vision images by GC Snakes.

Based on the proposed experimental scheme for the geometric parameter measurement, the validation experiments for the GC Snakes are carried out. In accordance with the above test plan, stereo passive forging images are segmented using two methods, namely the GC Snakes and method in [1], by which the dimensions and positioning of a forging test part are measured in nine positions along the *X_m_*, *Y_m_* axis and one general direction in the measurement coordinate system, respectively. Based on the experimental scheme in Figure 23, the experimental process can be described as feature edge extraction, image segmentation, feature extraction and reconstruction, and geometric parameter calculation. Therefore, the collected grayscale images of the testing forging part serve as the input of the measurement process, as shown in Figure 26. On this basis, feature edges for the forging are extracted as initial control points to be subscribed in GC Snakes, and the target contours by segmentation are shown in Figure 27. Hence, the image segmentation method proposed in this paper can effectively separate the test piece from the background.

If the segmented target area is referred to as the forging feature area, in order to extract the feature points that reflect the geometric parameters of the forging accurately, it is necessary to extract features near the target contour and on the upper surface of the forging within the feature area *S_F_*, respectively. Based on the target contour of GC Snakes, a feature point set *F* is established in the circular area near the target contour, represented as

(24) $$F=\left\{P(i,j)|\bar{PC_{0}}\in \left(R_{C_{0}}(\theta_{C_{0}}(i,j))-\delta_{C_{0}},R_{C_{0}}(\theta_{C_{0}}(i,j))\right]\right\}$$

where *R_C_*_0_(*θ_C_*_0_) represents the segmented target contour, and *C*_0_(*x*_0_,*y*_0_) is the least squares center of the target contour. It is worth noting that in order to accelerate the segmentation efficiency meanwhile ensuring measurement accuracy, it is adequate to extract feature points within a circular area with a width of *δ_C_*_0_ near the target contour. Similarly, when reconstructing the upper surface of a forging, it is also necessary to establish a feature point set *F*_0_ in the upper surface area S_0_ of the forging, represented as

(25) $$F=\left\{P(i,j)|\bar{PC_{0}}\in \left(R_{C_{0}}(\theta_{C_{0}}(i,j))-\delta_{C_{0}},R_{C_{0}}(\theta_{C_{0}}(i,j))\right]\right\}$$

**Figure 28:**
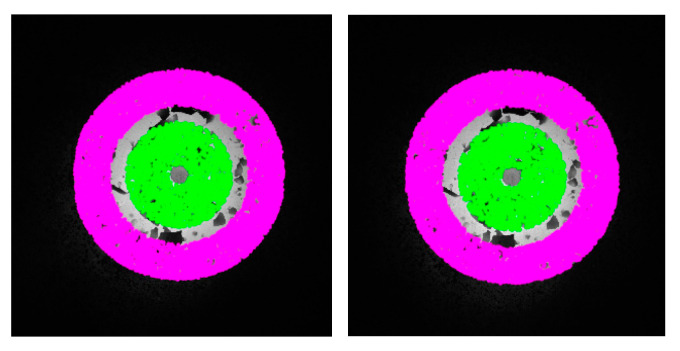
Feature points for the forging test part.

**Figure 29:**
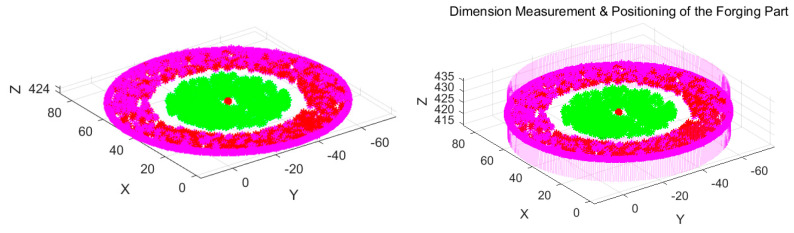
Reconstructed features and the envelope cylindrical surface.

Therefore, the features to be reconstructed consist of two parts during the validation experiments, namely the target contour features *F* and the upper surface features *F*_0_, as shown in Figure 28, where the pink points correspond to the target contour features *F* while the green points correspond to the upper surface features *F*_0_. The extracted forging features *F* and *F*_0_ are matched and then reconstructed in the measurement coordinate system, and the fit envelope cylindrical surface is built up to calculate the geometric parameters required, as shown in Figure 29.

**Figure 30:**
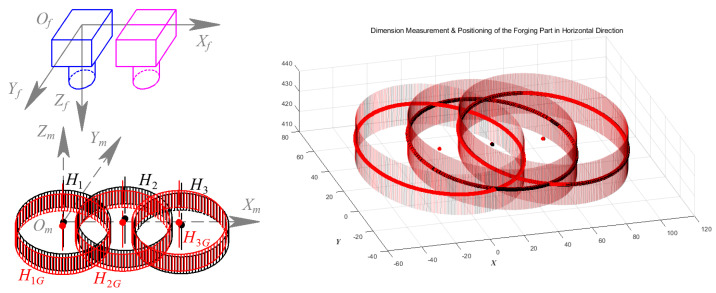
Geometric parameters measurement for forging part in the *X_m_* direction.

As is described by the test plan in Section 4.1, the validation experiments of the GC Snakes are operated by measuring dimensions and positions at nine measurement positions in three directions for the forging part. By establishing the measurements coordinate system {*O_m_*; *X_m_*, *Y_m_*, *Z_m_*} at the initial measuring position, Experiment 1 is conducted at three measurement positions set along the direction of the *X_m_* axis, as shown in Figure 30. Among the measurement results, H_1_, H_2,_ and H_3_ are the results of the segmentation method in [1], while H_1G_, H_2G_, and H_3G_ are those based on GC Snakes, respectively. The experimental results of the two methods are shown in Table 1 and Table 2; the maximum positioning error in the *X_m_* direction of the method in [1] is 0.62 mm, and the maximum dimension measurement error is 0.69 mm in diameter; while the GC Snakes segmentation method has a maximum positioning error of 0.53 mm in the *X_m_* direction and a maximum measurement error of 0.39 mm in dimension, which can meet the accuracy requirements for rough machining of forgings and is significantly superior to the method in [1].

**Figure 31:**
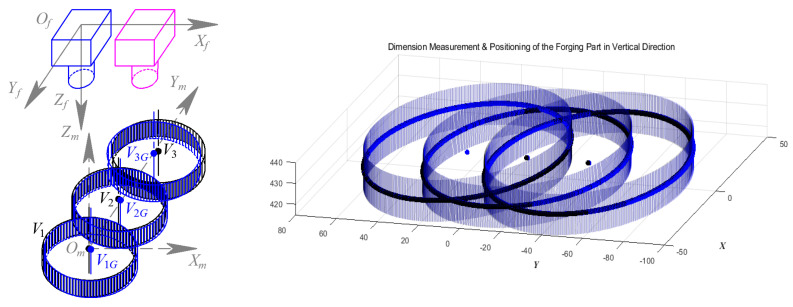
Geometric parameters measurement for forging part in the *Y_m_* direction.

Validation Experiment 2 is conducted at three measurement positions set along the direction of the *Y_m_* axis, as shown in Figure 31. Among the measurement results, V_1_, V_2,_ and V_3_ are the results of the segmentation method in [1], while V_1G_, V_2G,_ and V_3G_ are those based on GC Snakes, respectively. The experimental results of the two methods are shown in Table 3 and Table 4; the maximum positioning error in the *Y_m_* direction of the method in [1] is 0.79 mm, and the maximum dimension measurement error is 0.41 mm in diameter; while the GC Snakes segmentation method has a maximum positioning error of 0.55 mm in the *Y_m_* direction and a maximum measurement error of 0.37 mm in dimension, which can meet the accuracy requirements for rough machining of forgings and is also significantly superior to the method in [1].

**Figure 32:**
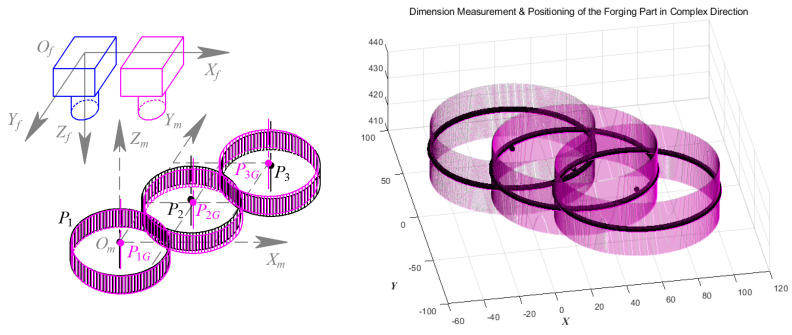
Geometric parameters measurement for forging part in a general direction.

Similarly, Validation Experiment 3 is conducted at three measurement positions set along a general direction, as shown in Figure 32. Among the measurement results, P_1_, P_2,_ and P_3_ are the results of the segmentation method in [1], while P_1G_, P_2G,_ and P_3G_ are those based on GC Snakes, respectively. The experimental results of the two methods are shown in Table 5 and Table 6; the maximum positioning error by the method in [1] is 0.71 mm, and the maximum dimension measurement error is 0.60 mm in diameter; while the GC Snakes segmentation method provides a maximum positioning error of 0.43 mm and a maximum measurement error of 0.23 mm in dimension, which can meet the accuracy requirements for rough machining of forgings and has been proven to be significantly superior to the method in [1].

### 4.3. Discussion and Limitations

In summary, the improved GC Snakes proposed in this paper can effectively improve the segmentation performance for forging images in real-time measurement and robustly solve the impact of grayscale distribution changes on segmentation results caused by working conditions. Regarding the external energy and force derived from the geometric properties of grayscale surfaces, GC Snakes is more percipient to the grayscale characteristics of forging images to improve the convergence for active contours. When GC Snakes is used to segment the forging images, initial control points are determined by feature edges, which envelop and contain the target forging part. However, it may result in unexpected segmentation performance if there exist outlier edging points caused by noise or interference involved in the initial control points. In subsequent algorithms, target recognition can be considered to be combined with GC Snakes to improve interference immunity and intelligence.

## 5. Conclusions

In this paper, an efficient and robust image segmentation model, namely the GC Snakes, as well as the corresponding image segmentation method is proposed for hot forgings in real-time measurement based on the geometric continuity of the equivalent grayscale surface of hot forging images. Originating from continuity parameters of grayscale surface, a new GC Snakes external energy and image force is defined, feature edging as the initial control points, and the image segmentation of hot forgings is achieved by minimizing the energy functional of the active contour curve. On this basis, image segmentation experiments using GC Snakes for grayscale images of forgings under different working conditions are conducted, and the segmentation performance is further verified through measurement experiments of the dimension and position for hot forging. The experimental results show that the proposed GC Snakes has satisfying performance for forging images with different grayscale distributions and can meet the accuracy and efficiency requirements for measurement of forgings with better stability and robustness compared to existing methods. As a result, GC Snakes has been proven to be suitable and has provided a theoretical basis for online real-time measurement in the processing of hot forgings.

In future works, GC Snakes and the corresponding image segmentation approach proposed in this paper are expected to be applied in the real-time image processing for grayscale and color images of various machine parts. With the image force and energy functional adjusted properly, the GC Snakes has promising prospects in medical image processing and analysis. Moreover, this study can also be integrated with other sorts of algorithms to realize further applications such as feature extraction, object detection, and image classification.

## Figures and Tables

**Table 1:** Experimental results in the *X_m_* direction for image segmentation method in [1].

Measurement Positions	Theoretical Position in the Forging Coordinate System	Relative Measurement Position in Camera Coordinate System *R_hi_* (*i* = 1, 2, 3)	Theoretical Positioning Distance/mm	Measurement Positioning Distance/mm	Real Dimension of Forgings Diameter/mm	Measurement Dimensions of Forgings Diameter/mm
H_1_	[0 0 0]	[0 0 0]	0	0	90.4	90.7509
H_2_	[30 0 0]	[30.6160 −0.4733 −0.3517]	30	30.6217		90.8470
H_3_	[60 0 0]	[60.4739 −0.7572 −0.3804]	60	60.4792		91.0868

**Table 2:** Experimental results in the *X_m_* direction for GC Snakes method.

Measurement Positions	Theoretical Position in the Forging Coordinate System	Relative Measurement Position in Camera Coordinate System *R_hi_* (*i* = 1, 2, 3)	Theoretical Positioning Distance/mm	Measurement Positioning Distance/mm	Real Dimension of Forgings Diameter/mm	Measurement Dimensions of Forgings Diameter/mm
H_1G_	[0 0 0]	[0 0 0]	0	0	90.4	90.6685
H_2G_	[30 0 0]	[30.5308 −0.2694 −0.3371]	30	30.5338		90.7868
H_3G_	[60 0 0]	[60.2630 −0.5892 −0.3598]	60	60.2670		90.7063

**Table 3:** Experimental results in the *Y_m_* direction for image segmentation method in [1].

Measurement Positions	Theoretical Position in the Forging Coordinate System	Relative Measurement Position in Camera Coordinate System *R_vi_* (*i* = 1, 2, 3)	Theoretical Positioning Distance/mm	Measurement Positioning Distance/mm	Real Dimension of Forgings Diameter/mm	Measurement Dimensions of Forgings Diameter/mm
V_1_	[0 0 0]	[0 0 0]	0	0	90.4	90.8085
V_2_	[0 −30 0]	[−0.6324 −29.2531 −0.2785]	30	29.2613		90.8108
V_3_	[0 −60 0]	[−0.7403 −59.2078 −0.1712]	60	59.2127		90.7786

**Table 4:** Experimental results in the *Y_m_* direction for GC Snakes method.

Measurement Positions	Theoretical Position in the Forging Coordinate System	Relative Measurement Position in Camera Coordinate System *R_vi_* (*i* = 1, 2, 3)	Theoretical Positioning Distance/mm	Measurement Positioning Distance/mm	Real Dimension of Forgings Diameter/mm	Measurement Dimensions of Forgings Diameter/mm
V_1G_	[0 0 0]	[0 0 0]	0	0	90.4	90.6077
V_2G_	[0 −30 0]	[−0.3816 −29.4437 −0.2760]	30	29.4475		90.7709
V_3G_	[0 −60 0]	[−0.4984 −59.7762 −0.1785]	60	59.7785		90.7072

**Table 5:** Experimental results in a general direction for image segmentation method in [1].

Measurement Positions	Theoretical Position in the Forging Coordinate System	Relative Measurement Position in Camera Coordinate System *R_pi_* (*i* = 1, 2, 3)	Theoretical Positioning Distance/mm	Measurement Positioning Distance/mm	Real Dimension of Forgings Diameter/mm	Measurement Dimensions of Forgings Diameter/mm
P_1_	[0 0 0]	[0 0 0]	0	0	90.4	90.6217
P_2_	[30 −30 0]	[29.5686 −29.4203 −0.1450]	42.4264	41.7118		90.8357
P_3_	[60 −60 0]	[59.6490 −59.4868 −0.0760]	84.8528	84.2418		91.0028

**Table 6:** Experimental results in a general direction for GC Snakes method.

Measurement Positions	Theoretical Position in the Forging Coordinate System	Relative Measurement Position in Camera Coordinate System *R_pi_* (*i* = 1, 2, 3)	Theoretical Positioning Distance/mm	Measurement Positioning Distance/mm	Real Dimension of Forgings Diameter/mm	Measurement Dimensions of Forgings Diameter/mm
P_1G_	[0 0 0]	[0 0 0]	0	0	90.4	90.5707
P_2G_	[30 −30 0]	[29.8767 −29.7600 −0.1112]	42.4264	42.1697		90.6259
P_3G_	[60 −60 0]	[59.7583 −59.6468 −0.0154]	84.8528	84.4332		90.6305

## Data Availability

The original data contributions presented in the study are included in the article; further inquiries can be directed to the corresponding author.
